# Solution Blow Spinning of Polyvinylidene Fluoride Based Fibers for Energy Harvesting Applications: A Review

**DOI:** 10.3390/polym12061304

**Published:** 2020-06-07

**Authors:** Rasheed Atif, Jibran Khaliq, Madeleine Combrinck, Ahmed H. Hassanin, Nader Shehata, Eman Elnabawy, Islam Shyha

**Affiliations:** 1 Department of Mechanical and Construction Engineering, Faculty of Engineering and Environment, Northumbria University, Newcastle upon Tyne NE1 8ST, UK; jibran.khaliq@northumbria.ac.uk (J.K.); madeleine.combrinck@northumbria.ac.uk (M.C.); islam.shyha@northumbria.ac.uk (I.S.); 2 Center of Smart Nanotechnology and Photonics (CSNP), Smart CI Research Center, Alexandria University, Alexandria 21544, Egypt; ahassan@ncsu.edu (A.H.H.); nader83@vt.edu (N.S.); eman.elnabawy@smartci.alexu.edu.eg (E.E.); 3 Department of Textile Engineering, Faculty of Engineering, Alexandria University, Alexandria 21544, Egypt; 4 Department of Engineering Mathematics and Physics, Faculty of Engineering, Alexandria University, Alexandria 21544, Egypt; 5 USTAR Bioinnovations Center, Faculty of Science, Utah State University, Logan, UT 84341, USA; 6 Kuwait College of Science and Technology (KCST), Doha District 13133, Kuwait

**Keywords:** SBS, PVDF, (nano)fibers, nanofillers, piezoelectricity

## Abstract

Polyvinylidene fluoride (PVDF)-based piezoelectric materials (PEMs) have found extensive applications in energy harvesting which are being extended consistently to diverse fields requiring strenuous service conditions. Hence, there is a pressing need to mass produce PVDF-based PEMs with the highest possible energy harvesting ability under a given set of conditions. To achieve high yield and efficiency, solution blow spinning (SBS) technique is attracting a lot of interest due to its operational simplicity and high throughput. SBS is arguably still in its infancy when the objective is to mass produce high efficiency PVDF-based PEMs. Therefore, a deeper understanding of the critical parameters regarding design and processing of SBS is essential. The key objective of this review is to critically analyze the key aspects of SBS to produce high efficiency PVDF-based PEMs. As piezoelectric properties of neat PVDF are not intrinsically much significant, various additives are commonly incorporated to enhance its piezoelectricity. Therefore, PVDF-based copolymers and nanocomposites are also included in this review. We discuss both theoretical and experimental results regarding SBS process parameters such as solvents, dissolution methods, feed rate, viscosity, air pressure and velocity, and nozzle design. Morphological features and mechanical properties of PVDF-based nanofibers were also discussed and important applications have been presented. For completeness, key findings from electrospinning were also included. At the end, some insights are given to better direct the efforts in the field of PVDF-based PEMs using SBS technique.

## 1. Introduction

**Figure 1:**
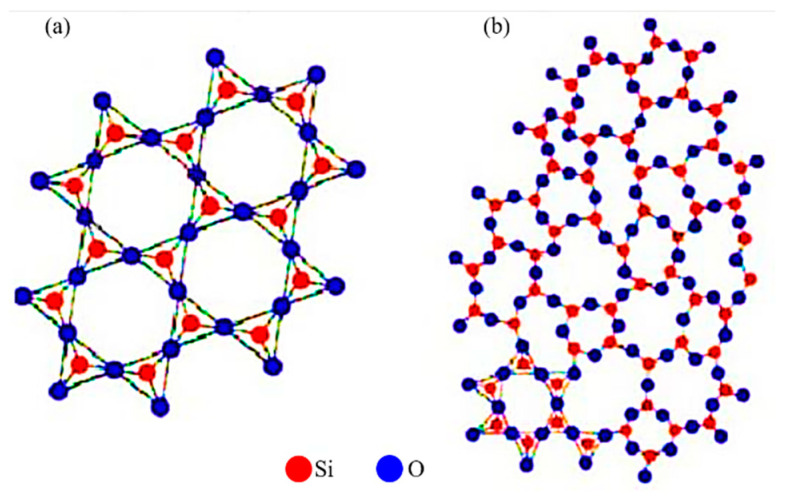
(**a**) Quartz SiO_2_ and its (**b**) amorphous crystal structure [2].

**Figure 2:**
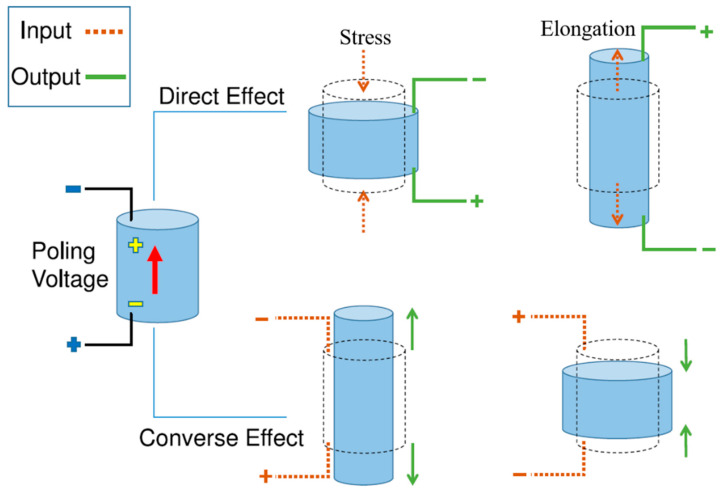
Direct and converse piezoelectric effects [4].

In 1880, Jacques and Pierre discovered that certain materials can generate electrical energy when subjected to mechanical strain through a phenomenon called piezoelectricity, and the materials exhibiting this characteristic are called piezoelectric materials (PEMs) [1]. One of the naturally occurring PEMs is quartz whose crystalline and amorphous structures are shown in Figure 1a,b, respectively [2]. There are two kinds of piezoelectric effects: direct and converse [3] In direct piezoelectric effect, voltage is generated at the application of mechanical strain while in converse piezoelectric effect, mechanical strain is generated upon the application of voltage as schematically shown in Figure 2 [4]. Piezoelectric efficiency is generally measured in terms of piezoelectric charge constant (d_ij_) (C/N) which is defined as the amount of charge density (C/m^2^) generated upon the application of mechanical stress of 1 N/m^2^ [5]. The subscripts *i* and *j* tell the direction of applied force and orientation of dipoles, respectively. The piezoelectric charge constant is related to piezoelectric voltage constant (g_ij_) (Vm/N or m^2^/N) and is given by $g_{ij}={\left(\varepsilon^{T}\right)}^{-1}d_{ij}$, where $\varepsilon^{T}$ is permittivity under constant stress $\vec{T}$ [6]. The absolute permittivity of the material ε (F/m) is given by $\varepsilon =\varepsilon_{r}\varepsilon_{0}$, where $\varepsilon_{0}$ is permittivity of free space (8.854 × 10^−12^ F/m), and $\varepsilon_{r}$ is dielectric constant (or relative permittivity) of the material [6].

**Figure 3:**
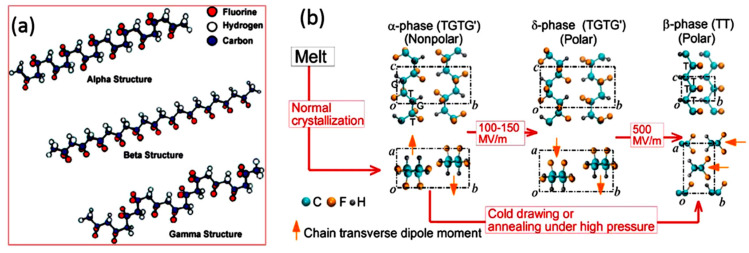
(**a**) Primary polymorphs of PVDF (α, β, γ). (**b**) Electric field-induced phase transitions of PVDF. The transverse dipole moment of each polymer chain is shown using an orange arrow that points from the negatively charged fluorine atoms to the positively charged hydrogen atoms. T-trans; G-gauche [28].

PEMs can be embedded into the final products of daily use, for example, gas sensors, pressure sensors, parking sensors, and piezoelectric motors and mobile phones [7,8,9]. Although most used PEMs are ceramic-based, however, due to their brittleness and high density, they are not ideal candidates for applications demanding flexibility such as flexible electronic screens [10,11]. In 1969, polyvinylidene fluoride (PVDF) was first reported as thermoplastic polymer PEM exhibiting the piezoelectric activity [12]. Different polymorphs of PVDF on the basis of repeating units of -CH_2_-CF_2_- are α, β, γ, δ, and ε, and are shown in Figure 3 [13,14]. The different phases are based on chain conformations; all-trans (TTTT) for β-phase, TGTG (trans-gauche-trans-gauche) for α and δ, and T_3_GT_3_G for γ and ε [15]. Generally, PVDF exists as α-phase which is non-polar due to random alignment of hydrogen and fluorine ‘dipoles’, γ- and δ-phases are weakly polar as they exhibit some alignment of so-called dipoles [16]. The β-phase displays the best piezoelectric and ferroelectric properties as all-trans chains cause all dipoles to orient in one particular direction giving a piezoelectric response [17]. Various ways have been reported to enhance β-phase such as annealing [18], solution casting [19], and spin coating [20]. It has also been reported that the β-phase can be obtained directly by high-temperature quenching from a melt or by casting from dimethyl acetamide (DMAc), a strongly polar solvent [21]. 

**Figure 4:**
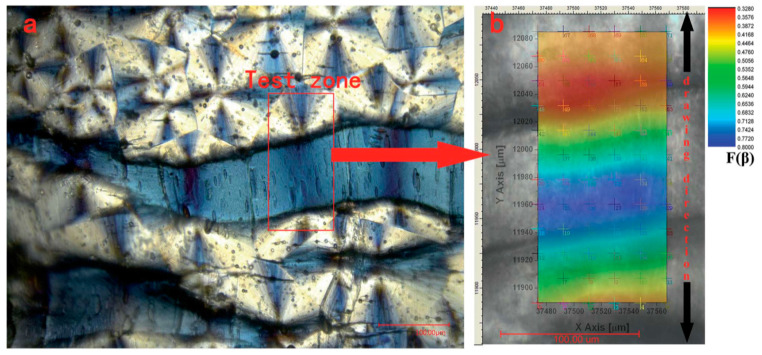
3D digital microscope observation and infrared microscope scanning of the PVDF samples after being stretched at 100 °C temperature and 1 mms^−1^ stretching rate. (**a**) The polarized photo of stretched samples observed by polarized module of 3D Digital Microscope; (**b**) the corresponding contour chart of F(β) of samples calculated from IR scanning [24].

The most widely used method to nucleate β-phase is either by mechanical stretching in uniaxial direction or by the application of high electric field [22]. However, it has been shown that fraction of β-phase increases mainly due to stretching and minimally due to electric field [21]. Uniaxial stretching tends the polymer chains to orient themselves and charge neutrality favors H and F atoms to segregate on opposite sides of the polymer chain resulting in piezoelectric β-phase [23]. As the stretching rate increases, fraction of α-phase decreases while β and γ phases dominate [23]. The fraction of β-phase saturates at a stretching rate of ~50 mm/min while the α-phase completely disappears at a ~600 mm/min [23]. In situ observation during uniaxial stretching shows that the deformation of the crystalline structure begins from the middle of α-spherulite and extends to one after another resulting in large-scale transformation from α to β phase [24]. Li et al. [24] carried out in situ microscopy as shown in Figure 4. They reported that stretching temperature (T_s_) can influence phase transformation and recommended a temperature of 100 °C [24]. 

PVDF-based PEMs are classified as stimuli responsive materials and have been employed as standalone or as matrices in composites and layered structures to fabricate stimuli responsive systems for applications such as drug delivery and tissue engineering [25]. One of the applications of PVDF-based PEMs is intelligent clothing to sense user activities in sports and personalized health care [26]. Various fabrication methods have been employed to produce fibers, such as gas jet spinning, nozzle-free centrifugal spinning, rotary jet spinning, melt blow spinning and flash-spinning. Out of all these, electrospinning has been extensively used for the fabrication of fibers; however, it has some limitations. Firstly, it can only be used for systems that are electrically conductive to conduct voltage applied during electrospinning process, and secondly, formation of a high fraction of β-phase is dependent on very high electric field making the process a safety hazard [27]. As there is electric field involved, it also requires the use of conductive collectors. It also has low yield making it a laborious process and unfit for scale-up.

**Figure 5:**
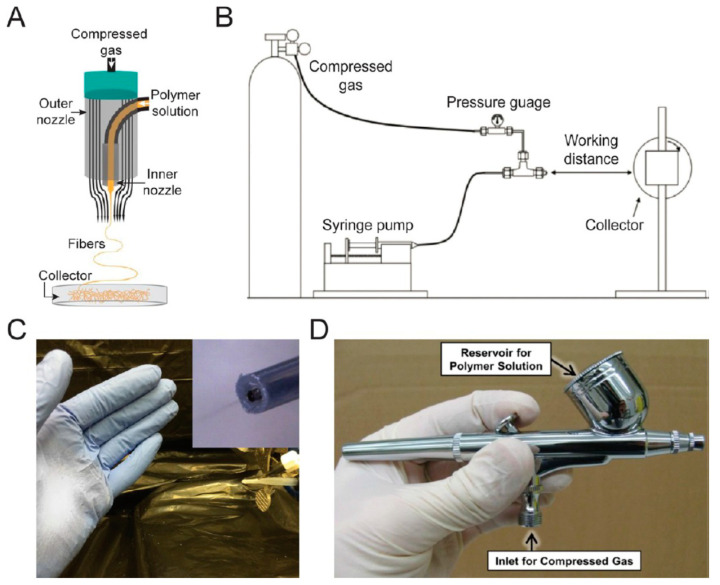
Various parts of SBS setup [29]: (**A**) Inlets for polymer solution and air with fibers coming out of nozzle due to attenuation force applied by high speed air [43]. (**B**) Schematic of SBS setup [44]. (**C**) Image of direct deposition of poly(styrene-block-isoprene-block-styrene) block copolymer fibers using a homemade solution blow spinning device [43]. (**D**) Commercial airbrush used for solution blow spinning [45].

Solution blow spinning (SBS) has emerged as an alternative technique to produce sub-micron/nano sized fibers and can relieve the user of the limitations posed by electrospinning. In SBS, polymeric precursor is dissolved into a suitable solvent to reduce its viscosity as thin fibers cannot be produced with very viscous polymer melt. The solution is then injected through a nozzle which is surrounded by a concentric outer pipe from which air is purged out. The solution interacts with the air and forms short fibers which fall on a collector. The photos and schematic of various components and setup are shown in Figure 5 [29]. The advantage of SBS is that it can be applied to both electrically conducting and insulating systems and does not require the application of electric field and conductive collectors to initiate fiber processing. Moreover, yield of fiber production is very high making it suitable for industrialization [30]. Parameters that influence the fiber production using SBS are discussed in the next sections.

## 2. Nanofillers

**Figure 6:**
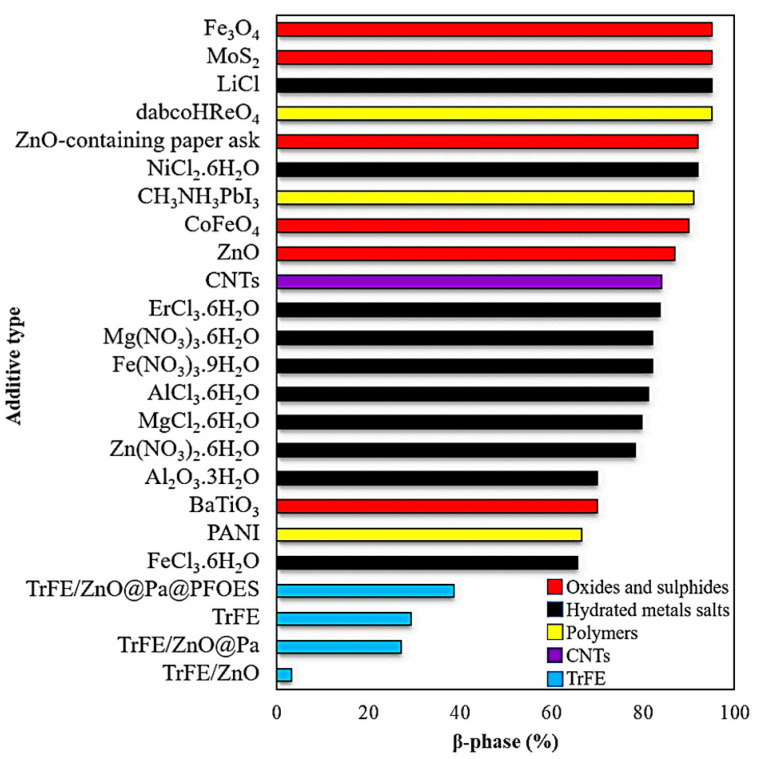
Fraction of β-phase obtained with different additives and copolymers [41,46,47,48,49,50,51,52,53,54].

The piezoelectric properties of neat PVDF are intrinsically lower than their inorganic counterparts [31]. One of the ways of enhancing piezoelectric properties is making copolymers. The copolymers of PVDF have chemical compatibility in high pH solutions, high impact strength at ambient and low temperatures and better clarity [32]. Some of the copolymers of PVDF include poly(vinylidene fluoride-hexafluoropropylene) [P(VDF-HFP)] and poly(vinylidene fluoride-trifluoroethylene [(P(VDF-TrFE)] [33]. It has been reported that the addition of trifluoroethylene (TrFE) in PVDF can promote the formation of β-phase due to the steric hindrance effect [34]. However, copolymers have been thoroughly reviewed [35] and will not be further discussed in this review. As inorganic PEMs have very high piezoelectricity, inorganic materials have been commonly incorporated in PVDF to enhance its piezoelectric properties [36]. The fraction of β-phase obtained using different nanofillers is shown in Figure 6. The nanofillers act as heterogeneous nucleation sites for β-phase and a hindering agent for the α-phase [37]. When a nanofiller is placed between the isolated polymer chains, it forms micro-capacitor structures due to interfacial interactions [38,39]. It increases the local electric field that promotes both migration and accumulation of charge carriers at the interface [38,40]. This interfacial polarization that causes the enhancement of a dielectric constant, is explained by Maxwell-Wagner-Sillars (MWS) effect [38]. However, it should be noted that not all nanofillers can enhance fraction of β-phase and some might inhibit the formation of β-phase. The incorporation of hydroxyapatite (HA) decreased the crystallinity and fraction of β-phase in HA/PVDF nanofibers [30]. Similar results were reported by Li et al. [41] where fraction of β-phase significantly dropped with unmodified zinc oxide (ZnO) nanoparticles mainly due to their agglomeration. The hybrid nanofillers produce synergistic effects in polymers that are useful to improve mechanical properties; however, when ZnO nanorods and graphene nanoplatelets were incorporated into PVDF along with hydrated metal salts, a drastic reduction in d_33_ was recorded [42]. It can be because of nanofillers assuming a competitive role with respect to H-bond formation between PVDF and the dissolved metal salt.

**Figure 7:**
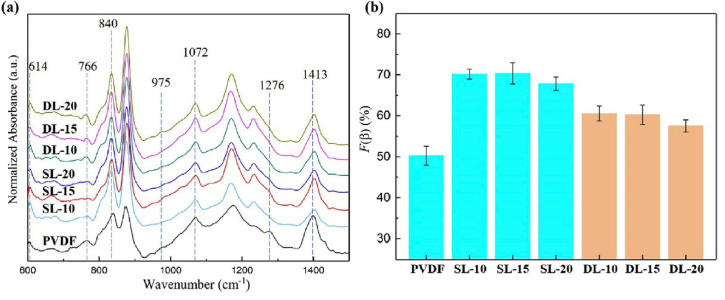
(**a**) FT-IR spectra and (**b**) concentration of β-phase at different concentrations of BaTiO_3_ [46].

The agglomeration of nanofillers is deleterious to the mechanical properties of polymer-based nanocomposites [55]. It is further reported that agglomeration of nanofillers is not beneficial to achieve a higher fraction of β-phase. The FT-IR spectra and β-phase content of single-layer (SL) and double-layer (DL) samples of PVDF/BaTiO_3_ nanocomposites are shown in Figure 7. The 840 cm^−1^ band, which relates to stretching of CF_2_ and C-C bonds, corresponds to the β-phase. The 880 cm^−1^ band, which could be ascribed to the rocking of C-C skeleton vibration, corresponds to non-polar α-phase [56]. The content of α-phase and β-phase is usually determined by the area ratio of 840 cm^−1^ band and 880 cm^−1^ band. The β-phase can be quantified using Beer–Lambert law as shown in Equation (1) [57];

(1) $$F_{\beta }=\frac{A_{\beta }}{\left(\frac{K_{\beta }}{K_{\alpha }}\times A_{\alpha }\right)+A_{\beta }}$$

where $A_{\alpha }$ and $A_{\beta }$ are the absorption of α and β-phases, respectively, *K_α_* and *K_β_* are the absorption coefficients at the corresponding wave number, which are 7.7 × 10^4^ and 6.1 × 10^4^ cm^2^/mol, respectively. As can be seen, the fraction of β-phase decreased in both SL/DL samples when concentration of BaTiO_3_ increased beyond 15 vol%. Sultana et al. [48] incorporated methylammonium lead iodide (CH_3_NH_3_PbI_3_) (MAPI) into PVDF and reported that the fraction of β-phase initially increased with the incorporation of MAPI, saturated at 10 wt%, and then gradually started to decrease. Hoque et al. [47] also reported that when the concentration of hydrated metallic salts increased beyond certain value, the fraction of β-phase started to decrease. The dielectric constant increased initially and then decreased upon further increase in salt concentration caused by agglomerates hindering the free chain movement of PVDF matrix [58]. Hence, a suitable concentration of nanofillers must be incorporated to avoid agglomeration and a subsequent reduction in β-phase content.

### 2.1. Carbonaceous Nanofillers

Carbonaceous nanofillers have been most commonly employed to enhance the fraction of β-phase in PVDF and in general mechanical properties of polymer nanocomposites [59]. The carbon atoms in graphene have a zig-zag structure which matches the zig-zag structure of the β-phase and therefore can be a strong nucleating agent [60]. Graphene oxide (GO) was more effective in enhancing piezoelectric and pyroelectric properties of PVDF than graphene [61]. Achaby et al. [37] found that the α-peaks completely disappeared at the incorporation of 0.075 wt% GO and a solely β-phase was observed. They concluded that a 0.1 wt% of GO is sufficient to nucleate all PVDF chains into β-phase. 

The attachment of PVDF chains to the GO sheets is caused by the interaction between CF_2_ in PVDF and the -C=O and COOH groups of the GO (hydrophilic interactions) [62].

### 2.2. Metallic Oxides

**Figure 8:**
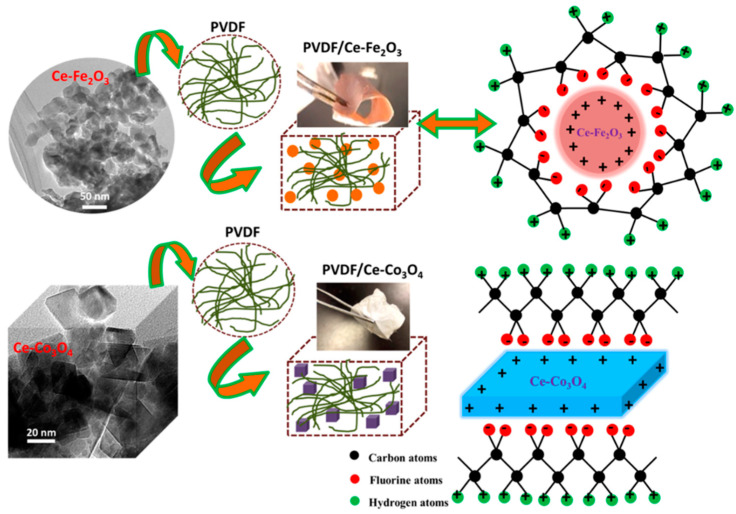
Schematic diagram of interaction between positively charged nanoparticles and dipoles in PVDF chains [63].

Metallic oxides, such as hematite (Fe_2_O_3_) and cobalt oxide (Co_3_O_4_), have also been employed as nanofillers [63]. PVDF nanofibers reinforced with 2 wt% Ce-Fe_2_O_3_ (Ce is cerium with atomic number 58) and Ce-Co_3_O_4_ recorded peak-to-peak output voltages of 20 V and 15 V with corresponding output currents of 0.010 and 0.005 µA/cm^2^, under the force of 2.5 N, respectively [63]. The transition metal cations influence the PVDF properties by affecting its chemical environment through covalent interactions as schematically shown in Figure 8 [63]. The nanofillers act as heterogeneous nucleation site and the positively charged surface of nanofillers attracts negative ends of PVDF dipoles. It results in the nucleation of electroactive crystalline phase. The growth of these nuclei is driven by electrostatic attraction-repulsion balance between dipoles. He et al. [64] also addressed the formation of electroactive phases by the electrical interfacial interaction between the positively charged organosilicate surface and the partially negative -CF_2_- bonds of the PVDF matrix. 

### 2.3. Hydrated Metal Salts

Hydrated metal salts have also been commonly incorporated into PVDF. The d_33_ and fraction of β-phase improve as the mean spherulite diameter of hydrated metal salts decreases [42]. A smaller particle exposes more surface per unit volume for β-phase to heterogeneously nucleate and is therefore more effective than a bigger particle. It was further observed that rare-earth ions such as Er^3+^ (Er is erbium with atomic number 68) are more effective nucleating agents for β-phase than transition metal ions such as Fe^3+^ (Fe is iron with atomic number 26) [47]. An enhancement in piezoelectric properties of PVDF by the incorporation of hydrated metal salts can be due to the large accumulation of surface charge between salt surface and PVDF matrix via MWS interfacial polarization [65]. A similar effect of interaction with the positively charged nanoparticles and the - CF_2_- dipoles of the PVDF chains, through which the stabilization of electroactive phase was achieved by Liu et al. [66]. Liang et al. [67] also suggested that the formation of electroactive phases in PVDF is due to the ion-dipole interactions between the positively charged molecules and -CF_2_- dipoles in PVDF or the negatively charged molecules and the -CH_2_- dipoles in PVDF chains. The piezoelectric properties degraded at higher fraction of nanofillers. Dhakras et al. [49] produced nickel chloride hexahydrate NiCl_2_.6H_2_O/PVDF nanofibers and reported that when the concentration of the nanofiller increased beyond 0.5 wt% the piezo-voltage dropped. The reason behind this drop could be the excess water content in the salt as it has been shown that excess water can notably affect the ferroelectric β-phase and in turn the electrical properties of the PVDF-based PEMs [20].

Fortunato et al. [42] incorporated hexahydrate metal salts of zinc (Zn), magnesium (Mg), aluminum (Al), and Fe into PVDF. They reported that the largest enhancement of piezoelectric charge constant (d_33_) and highest fraction of β-phase (82.17%) were achieved in case of magnesium nitrate hexahydrate Mg(NO_3_)_2_.6H_2_O. The increase in d_33_ can be attributed to the synergistic effect of the dipole moment associated with the nucleation of the electroactive phase and with the electrostatic interaction between the CF_2_ group of PVDF and the dissolved salt through hydrogen bonding. Magnesium has a highly negative standard redox potential ($Mg^{2+}+2e^{-}⇄Mg,-2.373\;eV$), which is similar to that of yttrium ($Y^{3+}+3e^{-}⇄Y,-2.372\;eV$) and very close to that of cerium ($Ce^{3+}+3e^{-}⇄Ce,-2.336\;eV$) [42]. The worst piezoelectric performance was observed in case of iron chloride hexahydrate FeCl_3_.6H_2_O [42]. It can be attributed to the relatively high mass and low negative standard redox potential of iron ($Fe^{3+}+3e^{-}⇄Fe,-0.037\;eV$), which weakens hydrogen bonding between PVDF chains and hexahydrate salts in polar solvents [42]. Hence, Mg(NO_3_)_2_.6H_2_O can replace cerium nitrate hexahydrate Ce(NO_3_)_3_.6H_2_O and yttrium nitrate hexahydrate Y(NO_3_)_3_.6H_2_O which are toxic salts.

### 2.4. Nanoclay

Nanoclays have also been incorporated into PVDF where the most widely studied clay is halloysite nanotubes (HNT). HNT is identical to kaolinite clay but has tubular morphology. Similar to montmorillonite, halloysite consists of two layers of aluminosilicate with Al:Si ratio of 1:1. The outer surface of HNT is made of Si-O units and the inner core comprises of Al-O units. Therefore, HNT has negative surface potential and partially positive potential from the inner core of HNT leading to the enhanced polymer solution conductivity [68]. HNT have been proven to act as a nucleating agent for PVDF, which is due to the dipole-dipole attraction between the oxygen atoms of HNT and C-H groups of PVDF. Alongside, the hydrogen bonding between hydroxyl groups of HNT and the fluorine atoms of PVDF enhances the formation of β-phase [69]. Khalifa et al. [70] incorporated HNT into PVDF and reported that HNT aligned themselves along the fiber axis and the produced nanofibers were fine, smooth, uniform and the mean fiber diameter decreased drastically with the incorporation of HNT. 

## 3. SBS Process Parameters

### 3.1. Molecular Weight

Two of the key factors that influence the viscoelastic properties of the polymer solution are molecular weight and molecular weight distribution (MWD). It has been shows that polymers with high molecular weights are more suitable for fiber spinning [71] and a higher molecular weight of precursor PVDF yield nanofibers with bigger diameters [72]. The molecular weight of precursor PVDF also affects the fraction of β-phase in the produced nanofibers [73]. When polymer has high molecular weight, the formation of bundles of fibrils is easy in the cross-linked polymer fiber [74,75]. At the evaporation of solvent, the polymer phase collapses laterally thereby resulting in a strong, dense and highly oriented fiber surrounded by an annulus of the solvent [74]. Gelation effects may render the fiber solid-like with indefinite lifetime in agreement with the literature [76,77]. 

### 3.2. Solvents

**Figure 9:**
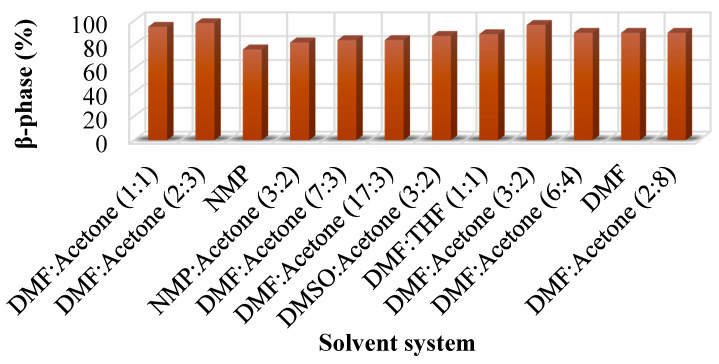
Various solvent systems used for PVDF-based PEMs and fraction of β-phase obtained [18,27,30,42,46,49,70,78,79,82,83,84,85,86,87,88,89].

Various organic solvents have been employed to dissolve PVDF and most commonly used is a mixture of N,N-dimethylformamide (DMF) and acetone [78]. Other solvents include N-methylpyrrolidone (NMP) [79], dimethyl sulfoxide (DMSO) [80], and tetrahydrofuran (THF) [81]. PVDF cannot be dissolved in THF. THF was used with DMF to dissolve PVDF [82]. As PVDF cannot be dissolved in THF, the answer of using THF with DMF to dissolve PVDF could not be found in the reviewed literature and is a potential gap in the available literature. Solvents reported in the literature and the resultant fraction of β-phase are shown in Figure 9. A mixture of DMF and acetone with different volume fractions has been mostly used. The highest fraction of β-phase (98%) was achieved when the solvent system was DMF:acetone (2:3) [27]. However, this information does not suggest to use DMF:acetone (2:3) to get maximum fraction of β-phase as there are various other parameters that influence the fraction of β-phase obtained and should be taken into account.

### 3.3. Evaporation Rate

Solvent evaporation rate is known to influence final diameter and fraction of β-phase in PVDF-based materials [90]. As a solvent is only used to dissolve polymer, it is not supposed to be a part of the final product. Therefore, its evaporation rate should be as high as possible. It can be explained on the basis of the law of conservation of mass. The mass of an unperturbed element of unit length in the straight part of the jet soon after it comes out of the nozzle decreases according to Equation (2) [91].

(2) $$\frac{d\left(fV\right)}{dx}=-h_{m}\left[C_{s,eq}\left(T\right)-C_{s,\infty }\right]2\pi a$$

where *f* is the area of a jet cross-section which is assumed to be circular of radius *a*, *V* is the absolute jet velocity, *x* is the axial coordinate reckoned along the straight jet axis, $C_{s,eq}\left(T\right)$ and $C_{s,\infty }$ are the solvent vapor volume fractions at the jet surface and far away from it, respectively, *T* is temperature which is the same for polymer solution and the surrounding air in case of SBS, $h_{m}$ is the mass transfer coefficient and is given by Equation (3) [91].

(3) $$h_{m}=\frac{D_{a}}{2a}0.495Re^{1/3}Sc^{1/2}$$

where $D_{a}$ is the solvent vapor diffusion coefficient in air, $R_{e}$ is Reynolds number given by [91],

(4) $$R_{e}=\frac{V2a}{\nu_{a}}$$

where $\nu_{a}$ is the kinematic viscosity of air, $S_{c}$ is is the Schimdt number and is given by [91],

(5) $$S_{c}=\frac{\nu_{a}}{Da}\;$$

The above equations suggest that the solvent vapor volume fraction at the jet surface should be high. This is possible when solvent has low boiling point and high evaporation rate. Dhakras et al. [49] added low boiling point solvent acetone into PVDF-DMF system and found that bead formation was significantly suppressed with the addition of acetone. Therefore, a solvent with low boiling point and high vapor pressure would be suitable for this purpose. Another probable way of increasing the evaporation rate is heating the polymer solution jet externally. 

### 3.4. Dissolution Method

#### 3.4.1. Manual Stirring

A simple way of dissolving PVDF in the solvent system is manual stirring. However, it is time-consuming and not suitable to disperse nanofillers as uniform dispersion of nanofillers requires prolonged and strong agitation. 

#### 3.4.2. Magnetic Stirring

In magnetic stirring, an electromagnet is used that continuously reverses its polarity under the application of AC voltage. Another regular magnet is placed in the beaker containing solvent and PVDF. The magnet rotates to keep its poles opposite to the electromagnet poles underneath the beaker. The magnetic stirring becomes slow or difficult when viscosity of the polymer solution increases. In addition, there is a possibility that solvent system may react and dissolve the polymer coating on the magnet. Hoque et al. [47] successfully dissolved 250 mg of PVDF and up to 20 wt% of erbium (III) chloride hexahydrate and iron nitrate (III) nonahydrate in 5 mL of DMSO at 60 °C under continuous magnetic stirring for 14 h. The addition of the salts led to vanishing of all XRD peaks corresponding to α and γ crystals while peaks at 2θ = 20.5 (110)/(200) became sharp indicating the nucleation and growth of β-phase [47].

#### 3.4.3. Sonication

One of the applications of PEMs is sonicator. A piezo crystal fluctuates under the application of AC voltage. The fluctuations generate ultrasonic waves that shake the PVDF-solvent system and PVDF gets dissolved. It is a very powerful method not only for dissolution but also to uniformly disperse nanofillers. Up to 20 vol% BaTiO_3_ nanoparticles were successfully dispersed in DMF by ultra-sonication to form PVDF/BaTiO_3_ nanocomposites [46]. 

### 3.5. Feed Rate

**Figure 10:**
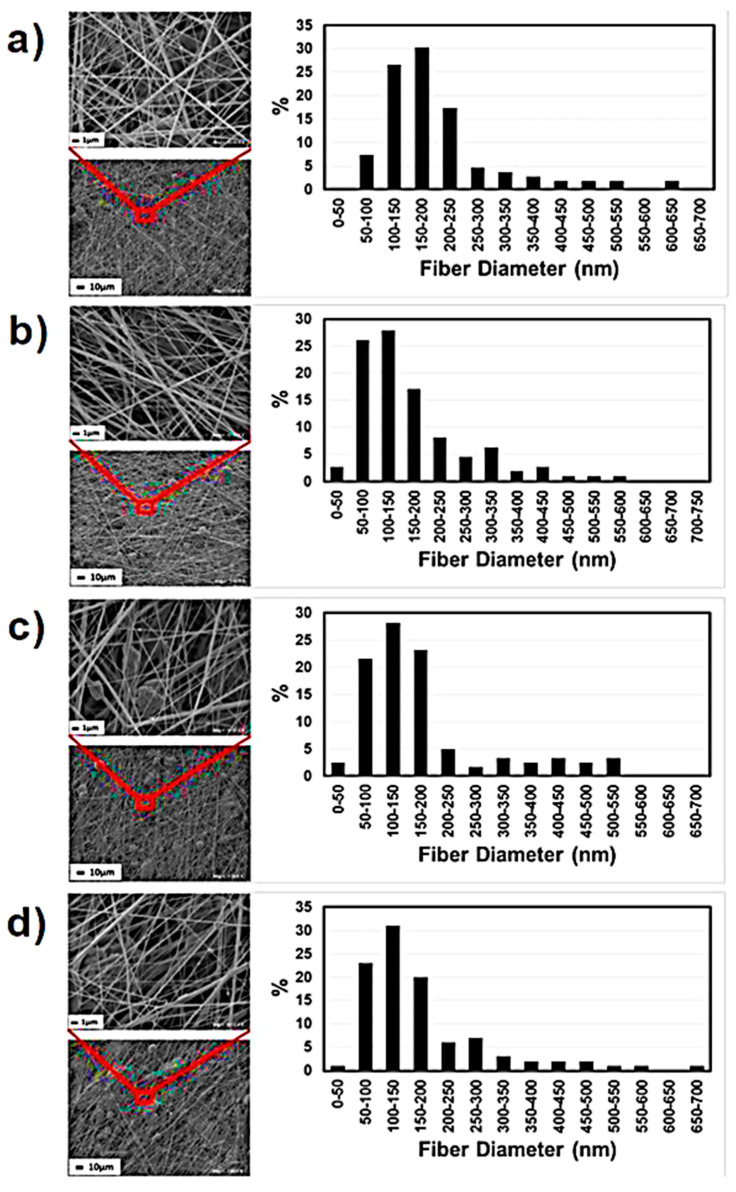
SEM images of solution blown thermoplastic polyurethane fibers with different feed rate values produced: (**a**) 1 mL/h, (**b**) 10 mL/h, (**c**) 25 mL/h, (**d**) 50 mL/h [92].

Mean fiber diameter increased with increasing feed rate of the polymer solution [92]. When feed rate is very high, solvent does not evaporate completely resulting in the formation of droplets on the web/collector [93]. To increase the evaporation rate of the solvent, Zhuang et al. [94] used a heating unit. Limited information about the influence of feed rate on the properties of PVDF-based nanofibers using SBS is available in the reviewed literature. Variation in fiber diameter of polyurethane with feed rate is shown in Figure 10 [92]. Polyurethane is used here as an example due to the similarity of rheological properties with PVDF [95]. At 1 mL/h, a majority of fibers are in the range of 100–250 nm. The range of fiber diameters lowered to 50–200 nm at 10 mL/h. It remained almost the same when feed rate increased to 25 mL/h. The fiber diameter slightly shifted to higher values at 50 mL/h. The maximum fraction of nanofibers with diameters up to 50 nm was achieved with 10 mL/h feed rate. Therefore, an optimum feed rate is essential to achieve the fibers with minimum diameter. 

### 3.6. Viscosity

The reviewed literature suggests a direct relationship between viscosity of polymer solution and mean fiber diameter. Haddadi et al. [96] incorporated hydrophobic and hydrophilic nanosilica into PVDF and reported that mean fiber diameter increased in both cases. They suggested that the viscosity of polymer solution increased by the incorporation of nanofillers which in turn led to an increase in mean fiber diameter. Yun et al. [32] fabricated Pb(Zr_0.53_Ti_0.47_)O_3_ reinforced PVDF nanofibers and reported that density and viscosity of the polymer solution increased after the incorporation of PZT. The mean diameter of nanofibers increased until 10 wt% and then started to decrease when volume fraction increased up to 30 wt% [32].

### 3.7. Weight Fraction

The weight fraction of parts should be selected such that there is no leftover of solvent as retained solvent causes degradation of mechanical properties by acting as stress concentration site [97]. Similarly, porosity in the PVDF nanofibers caused by fluids is deleterious to the mechanical properties [98]. If volume fraction of solvent is too low, the viscosity of the polymer solution will be high because of which a reasonable attenuation of polymer solution will be difficult to get thin fibers. On the other hand, a use of very large volume fraction of solvent will decrease the yield and increase the overall cost. Therefore, a minimum possible volume fraction of solvent should be used. 

### 3.8. Temperature

The processability of PVDF is easier because of its relatively low melting point (177 °C) and a glass transition temperature (T_g_) of −35 °C. PVDF solution temperature was reported to influence the spinnability of PVDF fibers [30]. The viscosity and temperature are inversely related. Therefore, low attenuation force will suffice to get thin fibers. Attenuation force in SBS is high speed air which means that thin fibers can be achieved at relatively low air pressure and velocity. On the contrary, when polymer solution is cold, its viscosity will be high and therefore high air pressure and velocity will be required to achieve thin fibers.

### 3.9. Air Pressure and Velocity

The air pressure has a significant impact on the morphology of the final product especially the fiber diameter [99]. Fiber diameter decreased and became more uniform with increasing air pressure. However, the fibers became defective when pressure was further increased [100]. When air is passed through the air inlet and moves toward the nozzle tip, it must be ensured that there is no choking [101]. A nozzle is choked when the maximum mass flow rate has been reached [102]. Any additional increment in pressure will result in an increase in chamber pressure. Internally the pressure might increase to a value in excess of the rated mechanical strength of the nozzle material which will result in catastrophic failure of the device. Externally of the nozzle an increase beyond choked conditions can lead to shock wave formation in the nozzle wake. The effect of shock structures on the fiber formation has not been determined; however, it is likely that the rapidly changing conditions before and after the shock will have a detrimental effect on the fiber morphology. To avoid choking, nozzle diameter, feed rate and air pressure must be carefully optimized [103].

**Figure 11:**
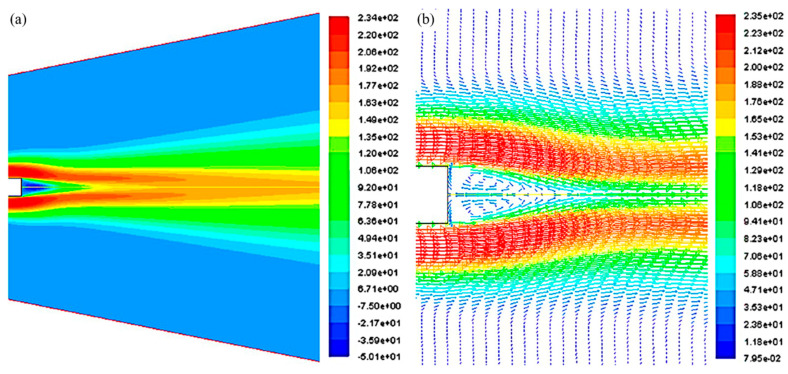
(**a**) Velocity contour. (**b**) Vector field in the vicinity of the nozzle end [100].

Computational methods have been employed to numerically investigate the influence of air characteristics on the fiber morphology in SBS [100,104]. Experimental studies that investigate airflow parameters for SBS are sparse. Figure 11 displays the velocity contour and vector plot for a typical subsonic nozzle. The flow presents with a recirculation zone of reversed flow directly behind the nozzle where the fiber is attenuated. Lou et al. [100] provided velocity plots along the centerline of the nozzle where flow reversal is observed. The flow velocity rapidly increases aft of the recirculation zone to a maximum value and then decreases monotonically. Turbulence intensities in the order of 40% are reported in the recirculation zone. To investigate the turbulent behavior of the flow, the k-ε turbulence model is one of the most commonly used models in CFD to simulate mean flow characteristics. This method results in rapid convergence [100] and is effective for solving problems involving reverse flow [105]. It is a semi-empirical model based on model transport equations for the turbulence kinetic energy (k) and its dissipation rate (ε). Neglecting gravitational effects, the transport equations for the k-ε turbulence model are given below [100];

(6) $$\frac{\partial \left(\rho k\right)}{\partial t}+\frac{\partial \left(\rho ku_{i}\right)}{\partial x_{i}}=\frac{\partial }{\partial x_{j}}\left[\left(\mu +\frac{\mu_{t}}{\sigma_{k}}\right)\frac{\partial k}{\partial x_{j}}\right]+2\mu_{t}\left(\frac{\partial u_{i}}{\partial x_{j}}+\frac{\partial u_{j}}{\partial x_{i}}\right)\frac{\partial u_{i}}{\partial x_{j}}-2\rho \varepsilon M_{t}^{2}$$

(7) $$\frac{\partial \left(\rho \varepsilon \right)}{\partial t}+\frac{\partial \left(\rho \varepsilon u_{i}\right)}{\partial x_{i}}=\frac{\partial }{\partial x_{j}}\left[\left(\mu +\frac{\mu_{t}}{\sigma_{\varepsilon }}\right)\frac{\partial \varepsilon }{\partial x_{j}}\right]+2C_{\varepsilon 1}\frac{\varepsilon }{k}\mu_{t}\left(\frac{\partial u_{i}}{\partial x_{j}}+\frac{\partial u_{j}}{\partial x_{i}}\right)\frac{\partial u_{i}}{\partial x_{j}}-C_{\varepsilon 2}\rho \varepsilon \left(\frac{\varepsilon }{k}+1\right)$$

where ρ is density kg/m^3^, k is turbulent kinetic energy m^2^/s^2^, *t* is time *s*, $u_{i}$ and $u_{j}$ are velocity fluctuations in the *i*th and *j*th directions, respectively, μ is viscosity kg/(m.s), $\mu_{t}$ is turbulent viscosity kg/(m.s), $\sigma_{k}$ and $\sigma_{\varepsilon }$ are turbulent Prandtl numbers for the kinetic energy and the dissipation rate, respectively, ε is dissipation rate of turbulent kinetic energy, $M_{t}$ is turbulent Mach number, $C_{\varepsilon 1}$ and $C_{\varepsilon 2}$ are parameters for k-ε turbulence model. There are numerous turbulence models available that can provide accurate results for this type of flow. This can be the subject of a future study.

### 3.10. Nozzle Design and Quantity

The flow of polymer solution is different from those of Newtonian fluids [106]. The stability of fiber spinning of polymer solutions at high tensile rates has been theoretically and experimentally studied for diluted polymer solutions while concentrated polymer solutions still need to be addressed [107,108,109,110]. The nozzle design is very critical in SBS as it significantly affects the airflow field distribution, air velocity and morphology of the final product [104]. Large diameter of the nozzle produced higher velocity which enhanced fiber attenuation and overall reduction in fiber diameter [104]. If the internal diameter of nozzle is too big, large droplets will be produced resulting in nanofibers with bigger diameters. Similarly, a very small orifice will reduce the throughput however it may produce fibers with small diameters. Once the process has been optimized to achieve maximum possible throughput, yield can be further increased by using an assembly of multiple nozzles and solution being injected through each nozzle simultaneously. A disk with 20 outlets for solution with two holes on the sides for compressed air was used to increase throughput [111]. They used the system to produce PVDF nanofibers with diameters in the range of 60–280 nm. The cumulative solution flow rate was 320 mL/h [111]. Other research groups have also tried to increase the throughput by using multiple nozzles [112] and grids [113]. 

### 3.11. Syringe Protrusion Length and Diameter

The influence of the protrusion length of the needle on fiber dimensions was found to be insignificant [104]. Lou et al. [100] also reported that the effect of protrusion length has insignificant effect on the fiber morphology. They used four different protrusion lengths: 4 mm, 2 mm, 0 mm, and −2 mm (minus sign means that the polymer syringe was retracted from the nozzle end by a distance of 2 mm). It was reported that the air velocity reaches a maximum in the vicinity of 10–20 mm below the nozzle face. The maximum air velocities were in the range of 170–180 m/s. However, based on lab experiments, the retracted nozzles resulted in intermittent process with polymer solution blocking the nozzle end. The protruded syringe was capable of producing fibers without such deficiencies. The best morphology of nanofibers was produced when polymer syringe was protruded out by 4 mm [100]. The diameter of the syringe will define the diameter of the droplet of the polymer solution. If the droplet diameter is large it can be potentially difficult to elongate it to get thinner fibers. It has been shown that a needle with a smaller diameter promotes fiber attenuation thereby resulting in thinner fibers [104]. 

### 3.12. Collectors

**Figure 12:**
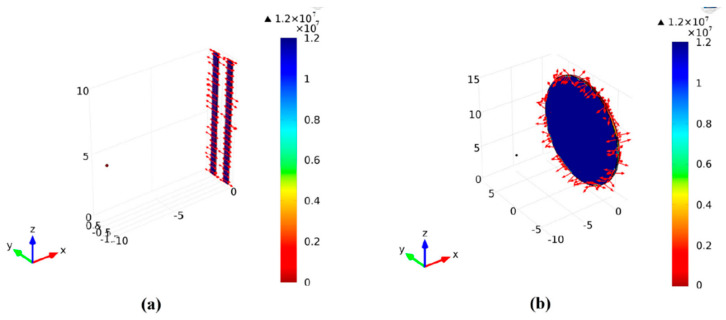
Electric field distribution for (**a**) two-bar collector, and (**b**) conventional collector [116].

Some commonly used collectors include copper wire drum [114], magnetic field [115], and two-metal bars to achieve statically aligned nanofibers [116]. Shehata et al. [116] demonstrated that two-metal bars as collector can significantly enhance the alignment of nanofibers compared with conventional dynamic technique in which a high speed rotating drum is used as collector. A comparison of the two types of collectors drawn by COMSOL Multiphysics package is shown in Figure 12 [116]. In the conventional collector, charge distribution is uneven that resulted in poor alignment of nanofibers. In contrast, an even charge distribution on two-metal bars resulted in efficient alignment of nanofibers.

## 4. Morphology of PVDF-Based Nanofibers

The morphological defects, such as beads, have deleterious effect on the piezoelectric properties [98]. Abbasipour et al. [98] reported that the output voltage in case of 0.8 wt% HNT/PVDF samples was higher than that of 0.8 wt% GO/PVDF samples even though the fraction of β-phase was higher in the latter samples. Hence, it is not just the fraction of β-phase but also morphology of nanofibers that influence the overall piezoelectric properties. 

### 4.1. Bead Formation

**Figure 13:**
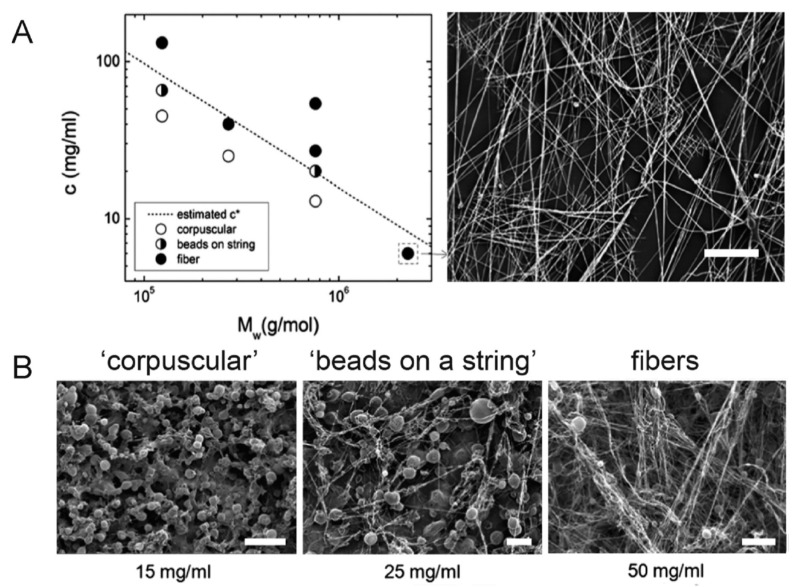
(**A**) Plot indicating morphology of poly(methyl methacrylate) (PMMA) sprayed using a solution blow spinning (SBS) apparatus at various concentrations and molecular weights. The estimated overlap concentration (c *) is indicated by the dashed line. Scanning electron microscopy (SEM) image of PMMA fibers formed at high molecular weight but below overlap concentration. Scale bar represents 50 μm. (**B**) SEM images of 50/50 wt. % PMMA/1H,1H,2H,2H-heptadecafluorodecyl polyhedral oligomeric silsesquioxane (PMMA: Mw = 593 kDa, PDI = 2.69) blends sprayed using an SBS apparatus at increasing concentrations of PMMA in solution. Scale bars represent 50, 100, and 50 μm, respectively [29].

The phenomenon of beads-on-string breakup of thin jets of dilute polymer solutions was discovered by Goldin et al. [117]. Its essence is that at later stages of capillary breakup “necks” between forming drops cease to thin and transform into thin liquid filaments gradually thinning without apparent change of shape of drops [118,119,120]. The presence of beads (Figure 13) may stem from a local higher concentration of polymer and charge accumulation [121]. The morphological defects in PVDF-based nanofibers can be suppressed by the incorporation of nanofillers, such as hydrated metal salts [49]. Dhakras et al. [49] produced PVDF nanofibers and reported that beads were observed on the produced fibers. When they incorporated nickel chloride hexahydrate (NiCl_2_.6H_2_O), the population of beads was found to decrease significantly. They further reported that the piezo-voltage increased up to 44% in neat PVDF when they were able to achieve beads free nanofibers [49]. Xin et al. [26] produced nanoclay/PVDF nanofibers and reported that the bead formation was suppressed by the incorporation of nanoclay.

### 4.2. Porosity

Porosity within PVDF fibers degrades the mechanical properties however it also increases the total charge collecting area and results in enhanced piezoelectric properties of PVDF-based PEMs [122]. Various techniques have been employed to increase through thickness macro-porosity in membranes, such as cryogenic spinning of fibers [123], laser drilling [124], sacrificial fibers [125], and in situ porosifiers to achieve interconnected macropores throughout the scaffold to improve cellular infiltration and enhance vascularization [126]. The densely packed membranes are also useful for many applications such as cell guidance substrates and in forming barriers in applications such as wound dressing and preventing infection (e.g., dental applications) [127]. The incorporation of LiCl results in increased porosity in PVDF nanofibers where long finger-like porosity was observed [128]. It was also observed that the average pore size decreased with increasing LiCl fraction [129].

### 4.3. Fiber Diameter

The mean fiber diameter and size distribution affect the properties and applications. For example, fibrous membranes are capable of generating different cellular response depending on fiber diameter [30]. Difference in fiber diameter influences the roughness and inter-fiber pore size of membranes and scaffolds used in tissue engineering applications and can have a direct influence on cellular adhesion, proliferation and differentiation [130]. Controlling fiber size is a strategy that can be used to tune pore size and mimic aspects of the extracellular matrix to alter cell infiltration [131]. This approach has been shown to enable the migration of human osteosarcoma cells (SaOs-2 cell line) from one side of a fiber membrane to the other, to support their proliferation [131]. The differentiation and spreading of osteoblastic cell line, MC3T3-E1 cell has also been reported to be affected by fiber diameter [132]. 

**Figure 14:**
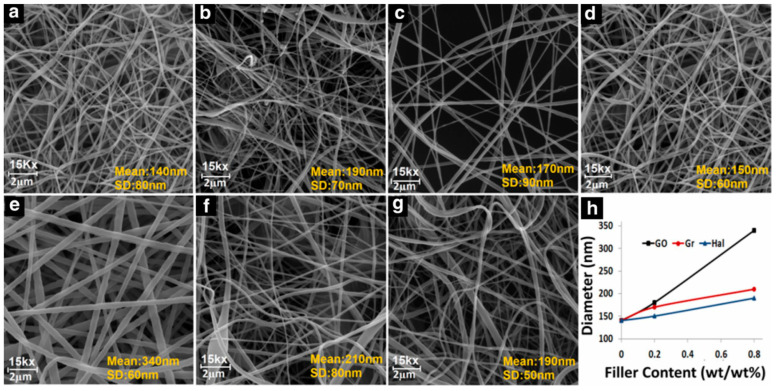
Morphology of PVDF-based nanofibers: (**a**) pristine PVDF, (**b**) 0.2wt%GO/PVDF, (**c**) 0.2 wt% graphene/PVDF, (**d**) 0.2 wt% HNT/PVDF, (**e**) 0.8 wt% GO/PVDF, (**f**) 0.8 wt% Gr/PVDF, (**g**) 0.8 wt% HNT/PVDF, and (**h**) variation in mean diameter with filler content [98].

The mean fiber diameter changes with the incorporation of nanofillers but contradictory results have been reported in the reviewed literature. Dhakras et al. [49] produced NiCl_2_.6H_2_O/PVDF nanofibers where the mean fiber diameter decreased with the incorporation of nanofiller. Khalifa et al. [133] incorporated nano alumina trihydrate (ATH) into PVDF and reported that mean fiber diameter decreased after the incorporation of ATH. On the contrary, Abbasipour et al. [98] reported that the mean diameter increased with different nanofillers as shown in Figure 14. The maximum increase in diameter in case of GO was due to interactions caused by hydroxyl and carboxyl groups of GO nanosheets [134]. Similarly, Fashandi et al. [135] produced cellulose nanocrystals/PVDF nanofibers and reported that the fiber diameter initially increased from 439 nm to 718 nm with the incorporation of 1 wt% cellulose nanocrystals. Upon further loading, fiber diameter decreased (552 nm at 3 wt% and 559 nm at 5 wt% cellulose nanocrystals) [135]. It should be noted that in all samples containing cellulose nanocrystals, fiber diameter is greater than neat PVDF fibers [135]. Tandon et al. [30] produced HA/PVDF nanofibers where mean fiber diameter increased after the incorporation of HA (~550 nm for neat PVDF and ~700 nm for HA/PVDF samples). The increase in fiber diameter with the incorporation of nanofillers can be attributed to increased viscosity and decreased solvent evaporation rate [136].

### 4.4. Alignment

**Figure 15:**
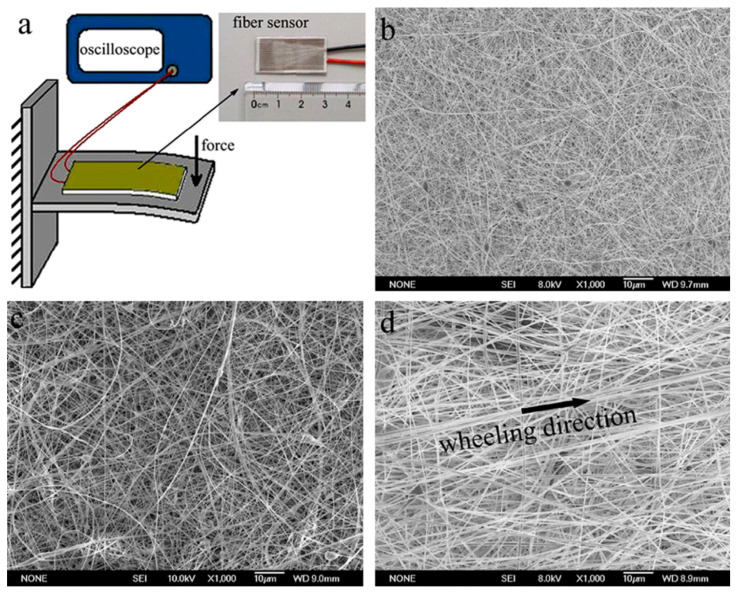
(**a**) Schematic illustration of the piezoelectric response experimental setup and the inset is photograph of the assembled full-fibre sensor; SEM images of (**b**) PVDF nanofibers, (**c**) PVDF/nanoclay nanofibers, and (**d**) PVDF/nanoclay nanofibers by NWS method [26].

The piezoelectric performance is affected by the preferential orientation of CF_2_ groups of PVDF [30]. Superior piezoelectric properties can be achieved by aligning the nanofibers in a particular direction [137]. A great deal of effort has been made to get the nanofibers aligned to enhance the piezo response [138]. Zaccaria et al. [137] produced random and aligned nanofibers of PVDF-TrFE. The electric response to mechanical stimuli, in the frequency range of 30–200 Hz is 2–4 times higher for aligned nanofibers compared with both random nanofibers and commercially available films. They further reported that an increase in piezoelectric response was due to the high fraction of β-phase in the aligned nanofibers. It was reported that the alignment also resulted in a reduction of mean fiber diameter [137]. Additionally, there was no delay between the electric response and the mechanical stress in case of aligned nanofibers while a remarkable phase shift was observed in case of random nanofibers. Abbasipour et al. [98] incorporated graphene, GO, and HNT into PVDF and reported that more oriented and finer nanofibers were achieved with HNT because of the tube-like morphology of HNT. To get aligned nanofibers, Xin et al. [26] reduced the nozzle-collector distance from normal value of 10–25 cm to only 3–5 cm and called it “near distance wheeling (NWS)”. They reported that this reduction in nozzle-collector distance helped in achieving aligned nanofibers as the average preferential fiber orientation got aligned with the collector rotation axis as shown in Figure 15 [26].

## 5. Mechanical Properties

**Figure 16:**
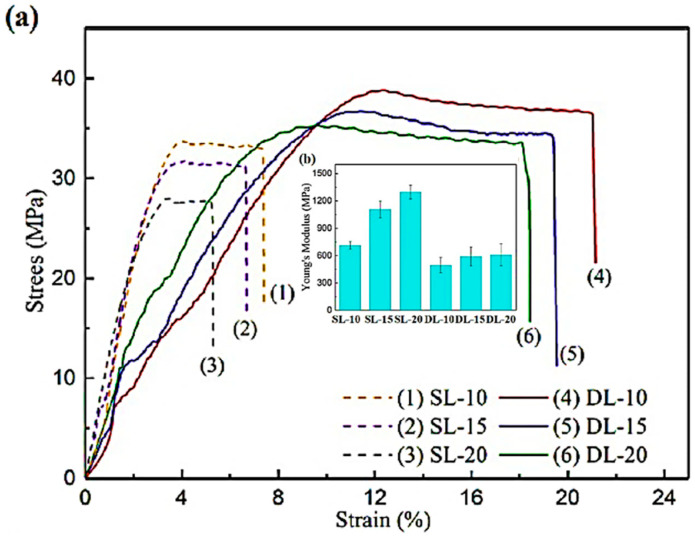
(**a**) Stress-strain curves, and (**b**) Young’s modulus of the PVDF/BaTiO_3_ nanocomposites [46].

As PEMs undergo cyclic loading, it is critical that they have superior mechanical properties. The fraction of β-phase, crystallinity, and morphology of nanofibers influence the mechanical properties. Tandon et al. [30] produced PVDF nanofibers using SBS and electrospinning, and reported that membranes obtained by SBS had higher tensile strength and lower Young’s modulus than those produced by electrospinning. β-phase does not only have superior piezoelectric properties but also has high elastic strength [139]. The incorporation of nanofillers can help improve mechanical properties of PVDF-based PEMs [46]. When BaTiO_3_ was incorporated into PVDF, not only fraction of β-phase but also mechanical properties improved as shown in Figure 16 [46]. At 10 vol% BaTiO_3_, ultimate tensile strength (UTS) increased in both single-layer (SL) and double-layer (DL) samples. The stress-strain curves suggest that PVDF initially shows ductile behavior with distinct elastic and plastic regions. At the onset of fracture strength, there is sudden drop in stress that suggests a very brittle fracture mode. However, mechanical properties degraded when concentration of nanofiller increased beyond 15 vol%. It is because when concentration is increased beyond a certain value, agglomeration takes place and agglomerates act as stress concentration sites [140]. Due to stress concentration, cracks initiate and propagate under the influence of cyclic loading and result in failure [141]. Therefore, agglomeration should be avoided to prevent degradation of mechanical properties. The enhancement of Young’s modulus degrades piezoelectric coefficient (d_33_) [142]. The d_33_ is defined as the change in polarization with applied uniaxial stress. At zero applied potential, d_33_ = - Pr/Y where Pr is remnant polarization and Y is Young’s modulus [142]. Hence, a PEM should have a lower Young’s modulus if a higher d_33_ (with minus sign) is required.

## 6. Applications

PVDF-based PEMs have found various applications including but not limited to energy conversion [143], power generation [144], sensing [145], and actuation [146]. Difference in fiber diameter influences the roughness and inter-fiber pore size of membranes and scaffolds used in tissue engineering applications and can have a direct influence on cellular adhesion, proliferation and differentiation [130,132,147]. Controlling fiber size is a strategy that can be used to tune pore size and mimic aspects of the extracellular matrix to alter cell infiltration [131]. This approach has been shown to enable the migration of human osteosarcoma cells (SaOs-2 cell line) from one side of a fiber membrane to the other, and support their proliferation [131]. The differentiation and spreading of osteoblastic cell line, MC3T3-E1 cell has also been reported to be affected by fiber diameter [132]. PVDF-based PEMs can be used in photocatalysis [148]. The spatial electric field of PVDF plays a generic enhancement role in the photocatalysis of both UV-light-responsive and visible-light-responsive photocatalysis [148]. In the presence of organic piezoelectric PVDF, the photocatalytic efficiency of a PVDF-TiO_2_ sample was improved by 55% and the corresponding first-order reaction rate constant increased by 5.42 times [148].

PVDF-based PEMs can also be employed where restricted wettability and hydrophobicity are desired such as to make water repellent coatings. PVDF is known to be a chemically resistant and hydrophobic polymer [30]. The fabrication process also affects the wettability [30]. Tandon et al. produced PVDF nanofibers via SBS and electrospinning, and reported that membranes produced by SBS had average contact angle of ~113° which was higher than those produced by electrospinning [30]. It suggests that PVDF nanofibers produced via SBS have lower wettability and a higher hydrophobic character than those produced by electrospinning. Hydrophobic character of PVDF fibers is explained in ref. [149,150,151]. PVDF-based PEMs are much promising in fabricating piezoelectric nanogenerators (PENGs) as the potential energy source for portable devices [152]. PVDF-based PEMs can also be employed to harvest energy from respiration and wind energy [50]. Alam et al. produced ZnO-containing paper ash ZPA/PVDF nanofibers-based PENG. They simply exhaled near a PENG and this mouth blowing led to generation of 0.2 V [50]. The further observed that the output voltage linearly increased from 0.2 to 1 V with an increase in mouth blowing wind flow from 1 ms^−1^ (corresponding to an exerted pressure of ~0.65 Pa) to 5 ms^−1^ (~16 Pa) [50]. The respiration process increases 4–8 folds during workout and therefore higher energy can be harvested during exercise [153]. This capability is ideal for harvesting energy from environmental wind flow or respiration making the PENG suitable for various applications, including charging mobile phones during conversations.

**Figure 17:**
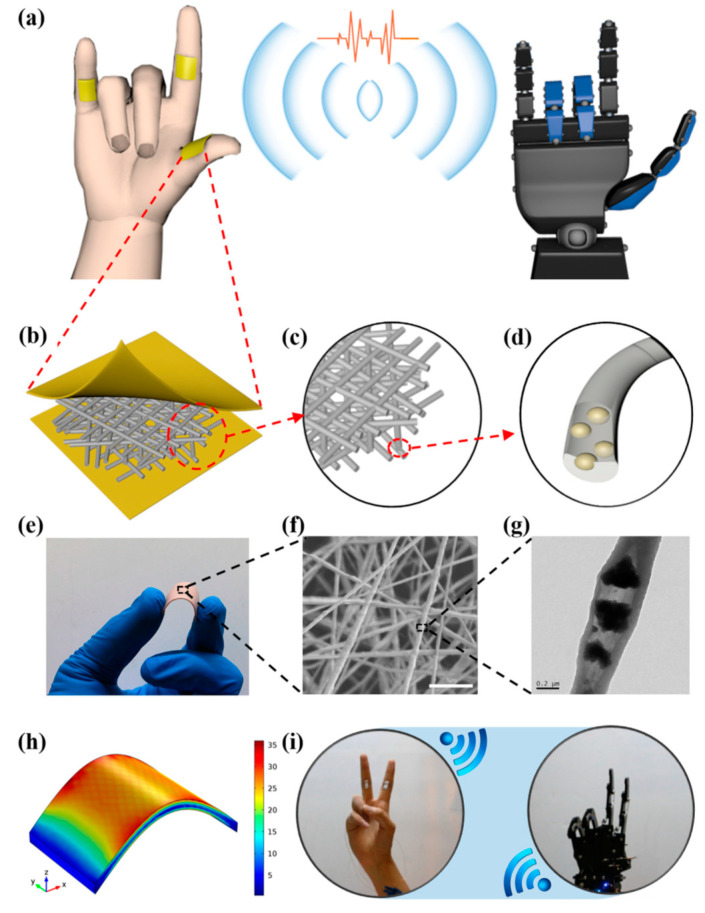
The structure design of the CPZNs-based self-powered PES. (**a**) The schematic of the developed smart sensor applied in the field of iHMI. The sketch of the device. (**b**) Nanofibers film. (**c**) The photograph of the fabricated sensor under bending mode. (**d**) Anatomy of sensor. (**e**) Photo of bent sensor. (**f**) The SEM image of the nanofibers. (**g**) The TEM image of a single nanofiber. (**h**) The result of the FEM simulation. (**i**) The application of robot hand remote control based on the PES [154].

Deng et al. successfully demonstrated that cowpea-structured PVDF/ZnO nanofibers (CPZNs)-based flexible self-powered sensors can be used to remote control of gestures in interactive human-machine interface (iHMI) [154]. The mechanism of the process is summarized in Figure 17 [154]. A robotic hand mimics a human hand based on the relationship between electrical output and the bending angle of the piezoelectric sensor (PES) (Figure 17a–g). The PES is attached to the inner knuckles of human fingers. The PES is comprising of PVDF/ZnO nanofibers mat and flexible MXene (Ti_3_C_2_) electrode. Due to the flexibility of PVDF-based nanofibers and electrode, the PES demonstrated good mechanical flexibility (Figure 17e). Upon the application of bending force, stress is concentrated in the middle region where tension/compression are maximum. The mechanical strain elicits piezoelectric response and voltage is generated at the far ends of the sensor (Figure 17h). When human hand gives a gesture of “Two” robotic hand replicates the gesture proving that bending sensing can be realized based on the piezoelectric effect. The sensitivity of PES could be regulated through the volume fraction of ZnO. The optimum bending sensitivity of 4.4 mV/deg with a fast response time of 76 ms could be achieved ranging from 44° to 122°. It has been shown that the output of PVDF-based motion sensors is gait sensitive [155]. This feature can help podiatrist to correct walking and running styles of patients and in forensic sciences for trace inspection.

## 7. Conclusions and Future Insights

PVDF is a multifunctional polymer, exhibiting piezoelectric, pyroelectric, ferroelectric and superior dielectric properties. To modify the piezoelectric properties of PVDF, its copolymers are made, and additives are incorporated. One of the most commonly used copolymers of PVDF is poly (vinylidene fluoride-co-trifluoroethylene) (PVDF-TrFE) because of high piezo response, lightweight and ease of processing, making it a potential candidate for flexible and wearable applications. In PVDF, there are five common phases; α, β, γ, δ, and ε. The phases are different based on chain conformations; all-trans (TTTT) for β-phase, TGTG (trans-gauche-trans-gauche) for α and δ, and T3GT3G for γ and ε. The phase obtained depends on the processing parameters. Among all, β-phase has the highest piezo response with highest dipolar moment and spontaneous polarization per unit cell. It is easier to achieve electrical polarization in semi-crystalline structure than in amorphous structure. Hence, higher the β-phase, greater the piezo response.

There are two potential methods to produce PVDF-based nanofibers namely electrospinning and solution blow spinning (SBS). The suitable fabrication method is the one which produces a higher fraction of phase with highest piezo response. To achieve a high piezo response, electrospinning requires the application of very high electric field (>100 MV/m) sometimes making the process a safety hazard. The advantage of electrospinning is that it combines mechanical stretching and electric poling into one process. SBS has many advantages over electrospinning. SBS is portable and nanofibers produced can be deposited on any substrate. One of the main advantages is throughput. SBS can give yield up to 30 times greater than that by electrospinning making SBS suitable for scale-up. Once nozzle design, feed rate, air pressure, solvent and polymer concentration are optimized, yield can be further increased by using an assembly of multiple nozzles and solution being injected through each nozzle simultaneously.

One of the challenges in SBS is reproducibility. Tandon et al. produced PVDF nanofibers via SBS and reported that SBS resulted in higher fiber variability between fabricated batches [30]. Mean fiber diameters of 400 ± 130 nm and 300 ± 130 nm were obtained for SBS and electrospinning, respectively [30]. The numbers suggest that SBS parameters need further optimization to achieve thinner fibers. Another aspect where SBS needs improvements is in the alignment of nanofibers. Tandon et al. produced PVDF nanofibers via SBS and electrospinning and reported that relatively poor alignment of nanofibers was achieved with SBS compared with electrospinning [30]. This could be due to increased turbulence around the collector because of high rotational speed and compressed air deflecting from the surface of the cylindrical collector [44].

Air pressure and velocity can significantly influence the fiber morphology. Although a higher centerline velocity helps reduce the fiber diameter, it is critical that the velocity does not reach supersonic to an extent where shocks may be generated as shocks can potentially break the fibers. During the literature review, no study was found that has predicted about whether shocks will actually break the fibers or will only result in sharp localized reduction in fiber diameter. Hence, a study is essential to investigate a threshold of a maximum air velocity and effects of shocks on the fiber morphology. Therefore, extensive research is still required to modify and optimize the SBS technique to produce PVDF-based nanofibers with superior piezoelectric properties. This modification and optimization require a confluence of both modeling/simulation and experimental research.
